# Rapidly growing double cancer of the gallbladder: A case report

**DOI:** 10.1016/j.ijscr.2023.108836

**Published:** 2023-09-17

**Authors:** Takamichi Suzuki, Hirokazu Matsuura, Hironobu Yamazaki, Satoshi Taguchi, Ayaki Koide, Takafumi Tabuchi

**Affiliations:** Department of Surgery, Kitaibaraki City Hospital, Japan

**Keywords:** Poorly differentiated adenocarcinoma, Gallbladder cancer, Double gallbladder cancer, No evidence of recurrence, Case report

## Abstract

**Introduction:**

Gallbladder cancer is a rare malignancy and double cancer of the gallbladder is extremely rare. Biliary tree malignancies including cholangiocarcinoma and gallbladder cancer are aggressive cancers and have a poor prognosis. This reports a rare case of double adenocarcinoma of the gallbladder.

**Presentation of case:**

A 77-year old woman with a cholelithiasis and decreased body weight was diagnosed with rapidly growing gallbladder tumor by abdominal computed tomography scan. A combined resection of the gallbladder, extrahepatic bile duct, segments 4a and 5 of the liver and regional lymph node dissection were performed. Pathologic examination revealed double poorly differentiated adenocarcinoma of the gallbladder. The patient had no evidence of recurrence for 40 months after resection.

**Discussion:**

This patient had double cancer of the gallbladder. Gallbladder cancer is an aggressive cancer and has a poor prognosis. The only curative therapy is radical resection. In this patient, radical laparotomy and adjuvant chemotherapy were performed. The pathological diagnosis of the resected specimen was double cancer of the gallbladder.

**Conclusion:**

This is a report of rapidly growing double cancer of the gallbladder. Patients with gallbladder cancer may benefit from aggressive surgical resection and adjuvant chemotherapy.

## Highlights

• Double cancer of gallbladder is rare.
• Extended surgery and adjuvant chemotherapy may improve survival in patients with high-risk features.

## 1 Introduction

Gallbladder cancer (GBC) is a rare malignancy but the most common malignancy of the biliary tract. GBC accounts for 80 %–95 % of biliary tract cancers worldwide [1]. GBC is also the most common cancer of the biliary tract in Japan [2]. Biliary tree malignancies including cholangiocarcinoma and GBC are aggressive cancers and have a poor prognosis.

About 90 % of gallbladder cancers are adenocarcinomas [3,4, but double cancer of the gallbladder is extremely rare. Synchronous cancers can occur from metastatic spread of an original cancer to a different site [5]. We describe a rare case of double cancer of the gallbladder in the present report.

## 2 Presentation of case

**Fig. 1:**
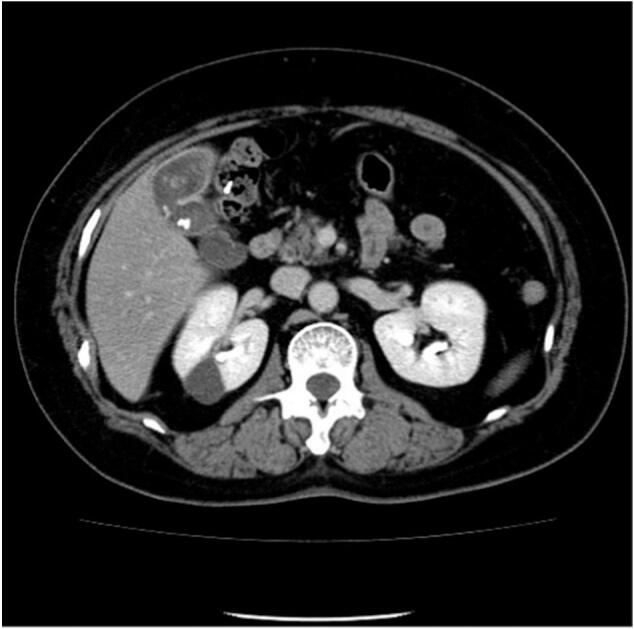
Computed tomography imaging taken 7 years before surgery.

A 77-year-old woman with a hypertension and a cholelithiasis presented to our hospital (Kitaibaraki City Hospital in Japan (public hospital)) with decreased body weight. She had a family history of lung cancer in her mother and sister. Gallbladder stones had been pointed out (Fig. 1). After 7 years she underwent a computed tomography (CT) scan due to suspected lung cancer.

**Fig. 2:**
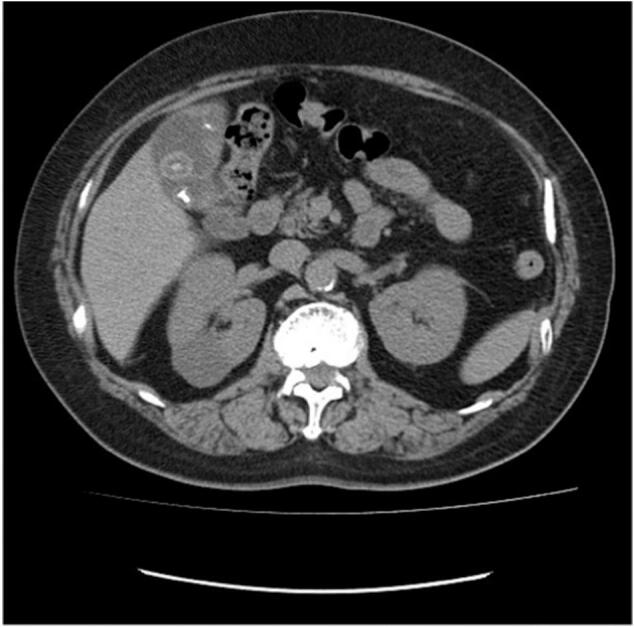
Computed tomography imaging taken 7 months before surgery.

A gallbladder tumor was suspected on the CT image (Fig. 2) and was advised further laboratory tests (for example tumor marker). But she believed that they were gallbladder stones rather than a malignant tumor. Her family persuaded her to have an imaging examination. But she did not get clinical examination. After 6 months she presented to our hospital. At this time, blood test and diagnostic imaging test were performed. No special abnormality was found on physical examination. Laboratory tests showed no abnormalities. Serum tumor markers were also within normal limits (CEA 1.8 ng/mL, CA19–9 20.0 U/mL).

**Fig. 3:**
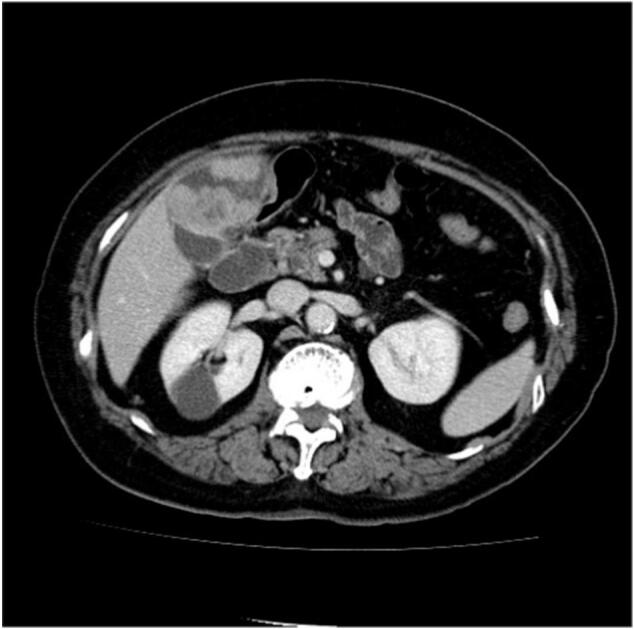
Computed tomography imaging taken 1 month before surgery.

Abdominal CT scan examinations showed that the tumor was clearly enlarged compared to CT image 6 months ago (Fig. 3). And CT showed lymphadenopathy adjacent to the common bile duct. We were diagnosed with double cancers of gallbladder with stones and regional lymph node metastasis. Under general anesthesia, cholecystectomy, extrahepatic bile duct resection, hepatic resection of segments 4a and 5 and regional lymph node dissection were performed.

**Fig. 4:**
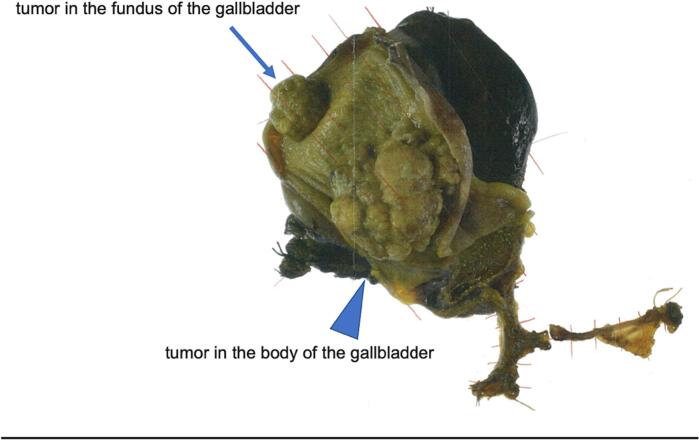
Postoperative specimen.

The gross specimen showed two tumors in the fundus and the body of gallbladder (Fig. 4). The patient made was discharged on postoperative day 24 although she had a surgical site infection.

**Fig. 5:**
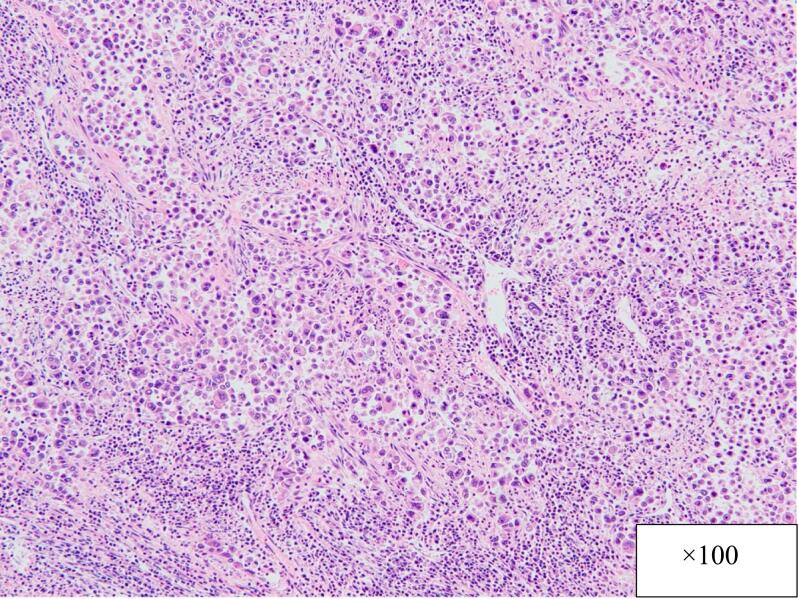
Postoperative specimen (the body of the gallbladder).

**Fig. 6:**
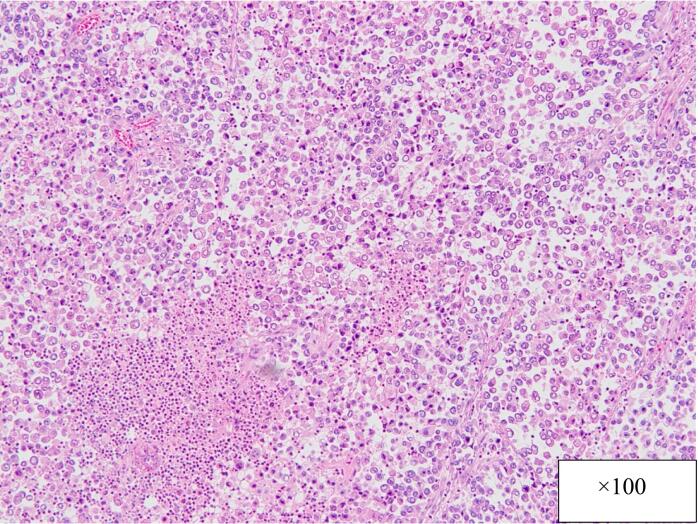
Postoperative specimen (the fundus of the gallbladder).

Postoperative pathology revealed adenocarcinoma of the gallbladder and a distinct transitional zone was observed between two tumors. The two tumors are very similar, adenocarcinoma (GnC, nodular-infiltrating type, 45 × 45 mm, por1, med, INFβ, pHinf0, pBinf0, pPV0, ly(0), v1, pT2(SS), pn0, pN0 DM(0), HM (0), EM (0)) in the body of the gallbladder (Fig. 5), adenocarcinoma (GnC, nodular-infiltrating type, 20 × 25 mm, por1, med, INFβ, pHinf0, pBinf0, pPV0, ly(0), v0, pT1b(MP), pn0, pN0 DM(0), HM (0), EM (0)) in the fundus of the gallbladder (Fig. 6), but the two tumors are clearly separated. It means double cancer of the gallbladder macroscopically and pathologically.

1-year adjuvant chemotherapy using S-1 was performed after discharge. The patient is currently seen in follow-up at an outpatient clinic, with no evidence of recurrence 40 months after resection.

## 3 Discussion

GBC is a rare but accounts for 80 %–95 % of the biliary tract malignancy. There is the wide variation of GBC incidence worldwide and 2 major groups of high-risk populations are identified in Latin America and in Asia [6]. GBC is also the most common cancer of the biliary tract in Japan [2].

The highest frequency of GBC is found among females over the age of 65 [7]. There is an association between chronic cholelithiasis and GBC. Gallstones or cholecystitis are found in more than 95 % cases of GBC [8]. A hyperplasia of the gallbladder evolves toward atypical hyperplasia, and this finally becomes invasive carcinoma [9]. The present patient is also over 65-year-old female and had a cholelithiasis.

GBC is mostly adenocarcinoma and 60 % originate in the fundus of the gallbladder, 30 % in the body, and 10 % in the neck [7]. Other types of GBC, like squamous cell carcinoma or adenosquamous cell carcinoma are also seen. But synchronous double gallbladder cancer is very rare. Most cases of synchronous cancer are reported from Japan and India [5]. In most of the case reports, the origin of double cancer of the gallbladder is within other organs such as liver, extra hepatic bile duct and pancreas. We represent a case of synchronous double gallbladder adenocarcinoma. Differentiating between synchronous cancer and metastasis is important. Therefore, we use criteria which include growth pattern, histologic differences between two tumors and lack of anatomical continuity between them. Gertsch P reports that there are clear histologic differences between the synchronous cancers [10]. In our case, both tumors in gallbladder are geographically distinct and a transitional zone is found microscopically between two tumors. Postoperative pathology revealed the very similar characteristics of the two tumors. We considered intra-organ metastasis more than synchronous cancers (or double primary cancer). However, there is room for debate regarding this consideration, and we think that there is insufficient evidence to make a clear conclusion.

Biliary tree malignancies including cholangiocarcinoma and GBC are aggressive cancers and have a poor prognosis. GBC confers a 5-year overall survival rate of approximately 20 % [11]. The poor prognosis is of GBC likely due to nonspecific symptoms and aggressive tumor biology that result in advanced stages at diagnosis. The main prognostic factor for GBC is the clinical or pathologic stage [12]. It is a matter of fact that more advanced cancer has a poorer prognosis. Lymph node metastasis is also important for the prognostic factor in advanced GBC [13].

The only curative therapy in GBC is radical resection with free margins. Surgical treatment is recommended as the first-line therapy for patients with resectable GBC [14]. In Japan, the 5-year survival rate of patients after the resection of advanced gallbladder cancer is 79 %–91 % for International Union Against Cancer (UICC) stage I, 64 %–85 % for stage II, 33 %–65 % for stage III, and 8 %–25 % for stage IV [13]. Although simple cholecystectomy seems to be enough for treating GBC at an early stage, radical surgery with hepatic resection and hilar lymphadenectomy has been considered the gold standard therapy in more advanced stages [15]. In advanced gallbladder carcinoma, a better survival was achieved in an aggressive surgical approach, in order of a S4a + S5 hepatic resection, an extended cholecystectomy and a cholecystectomy [16]. In our case, the tumor grew rapidly and was judged to be more malignant than expected. A case of T1a gallbladder carcinoma developed lymph node metastases has also been reported [17], so we decided to perform extended surgery. We performed cholecystectomy, S4a + 5 hepatic resection, extrahepatic bile duct, and regional lymph node dissection. Preoperative computed tomography showed lymphadenopathy adjacent to the common bile duct. Surgical findings revealed elastic hard lymph nodes about the size of a little fingertip, and extrahepatic bile duct resection was performed. Following R0 resection of stage T2 and higher N1 GBC, patients should be considered for adjuvant systemic chemotherapy [18], so we performed 1-year adjuvant chemotherapy using S-1 after discharge. We achieved a good result, and this patient has been followed up without evidence of recurrence for 40 months after resection.

## 4 Conclusion

GBC is rare and has a high mortality rate. Double cancer of gallbladder is even more rare. Patients with GBC may benefit from aggressive surgical resection. If GBC is advanced, favorable survival rate can be achieved after adjuvant chemotherapy.

## Consent

Written informed consent was obtained from the patient for publication of this case report and accompanying images. A copy of the written consent is available for review by the Editor-in-Chief of this journal on request.

## Methodology

The work has been reported in line with the SCARE guideline [19].

## Ethical approval

Ethical approval for this study (Ethical Committee notification number 0403) was provided by the Ethical Committee of Kitaibaraki City Hospital Ibaraki prefecture, Japan on 21 November 2022.

## Funding

N/A.

## Author contribution

Dr. Takamichi Suzuki is the main author and he has designed this report.

Dr. Hirokazu Matsuura participated in Conceptualization.

Dr. Hironobu Yamazaki participated in writing and Methodology.

Dr. Satoshi Taguchi, participated in Investigation and Term.

Dr. Ayaki Koide participated in Data curation.

Dr. Takahumi Tabuchi is the writer of this article and corresponding author.

## Guarantor

Takamichi Suzuki, Takafumi Tabuchi.

## Research registration number

Not applicable.

## Conflict of interest statement

N/A.
